# Study on the aggregation behavior of kaolinite particles in the presence of cationic, anionic and non-ionic surfactants

**DOI:** 10.1371/journal.pone.0204037

**Published:** 2018-09-13

**Authors:** Lingyun Liu, Liang Shen, Weirong Li, Fanfei Min, Fangqin Lu

**Affiliations:** Department of Materials Science and Engineering, Anhui University of Science and Technology, Huainan, China; VIT University, INDIA

## Abstract

Aggregation behaviors of kaolinite particles with different surfactants were studied in this paper. Aggregation settling yield and fractal dimension analysis were used to determine the aggregation results. Zeta potential measurements, adsorption tests, Infrared spectroscopy analysis and scanning electron microscope measurements were conducted for further investigation into the mechanism. Experimental results showed that much better aggregation results was obtained in the presence of cationic surfactant than that in the presence of anionic and non-ionic surfactants. 98% aggregation setting yield was obtained in the presence of dodecylamine. Adsorption tests indicated that the adsorption capacity of dodecylamine on kaolinite surface was larger than that of sodium oleate and Tween80. Zeta potential measurements confirmed that dodecylamine was more beneficial to the aggregation of kaolinite particles. Infrared spectroscopy analysis revealed that the adsorption of dodecylamine on kaolinite surface was attributed to electrostatic and hydrogen-bonding interactions. Sodium oleate was adsorbed by chemical adsorption. However, Tween80 can hardly be adsorbed by kaolinite surface.

## Introduction

The efficient treatments of fine tailings and waste water are easy to be underestimated in mineral processing process [1]. Tailings are inevitable by-product in the process of mineral mining and preparation. For coal preparation plant, slime water of tailings results in high solids concentrations and degradation of the coal washing circuit, which poses a severe challenge to the entire production process [2]. The interaction of fine particles with various coagulants and focculants are important in dewatering processes [3, 4]. The presence of the fine and ultrafine particles in the raw material causes the difficulty of separation, the loss of valuable components and ecological contamination [5]. Kaolinite is the main mineral component of clay deposits and a typical layered silicate mineral [6]. Due to the surface charge and strong hydrophilicity of kaolinite particles, there is a strong electrostatic repulsion and hydration repulsion between the particles, which is detrimental to the settling of fine minerals in dispersion medium and brings adverse effect on separation, sedimentation and clarification of waste tailing. The presence of fine kaolinite particles can exacerbate the copper flotation process [7]. In recent years, Xing et al. investigated the effects of kaolinite and montmorillonite on coal flotation in deionized water and calcium solution by flotation experiments, considering that the low flotation recovery and poor selectivity were obtained for coal-kaolinite system [8]. Consequently, the kaolinite particles were detrimental to the flotation process [9]. Simultaneously, the fine kaolinite is also one of the main industrial raw materials, and widely used in ceramics, electrical appliances, building materials, rubber, paper and other departments [10]. When the fine kaolinite is extracted from the suspension, it is conducive to the clean production of mineral processing and the fine kaolinite can be effectively utilized. However, it is a technical problem to remove fine kaolinite from metal-leaching or complex solutions. For this reason, the aggregation settlement characteristics and mechanism of fine kaolinite particles under different kinds of surfactant solution were studied in this paper, which could provide theoretical and technical support for aggregation separation of the fine kaolinite particles.

In the present study, a large number of studies investigated the dispersion of kaolinite particles in suspensions. For example, the mechanism of kaolinite suspension at different pH values was described using the EDLVO theory [11]. The hydrophobic aggregation of fine particles in high muddied coal slurry water in the presence of quaternary ammonium salts was investigated and it showed that quaternary ammonium salts can promote the settlement of coal slurry water [12]. The stability of colloidal kaolinite dispersions in the presence of dodecylamine (DDA) was studied, showing that DDA can induce a strong aggregation of colloidal kaolinite in aqueous suspensions [11]. Shen et al. investigated the flotation recovery of fine kaolinite when mixed dodecylamine chloride/fatty acid was used as collector, proving that high kaolinite flotation recovery was obtained in the presence of mixed dodecylamine chloride/fatty acid [13]. The variations of shear flocculation of colemanite mineral with sodium oleate (SO) and sodium dodecyl sulfate (SDS) were also investigated. It was determined that SO was more beneficial to the flocculation than SDS [14]. In addition, Liang et al. studied the effect of the polyaluminum chloride (PAC) on reducing the kaolinite entrainment on coal flotation [15]. Liu et al. investigated the effect of butanol on quartz flotation when N-dodecyl ethylenediamine (ND) was used as collector, single mineral (hematite or quartz) flotation and artificial mixed mineral (hematite and quartz) separation were conducted, indicating that butanol could improve the collecting performance of ND on quartz [16]. The flotation and adsorption behaviors of dodecyl trimethyl ammonium chloride (DTAC) and cetyl trimethyl ammonium chloride (CTAC) on diaspore and kaolinite were observed [17]. Although there are lots of researches on the effect of pH, alkylamine and different interface characteristics for the kaolinite aggregation settlement, few of them are about the difference of the effect of cationic, anionic and nonionic surfactants on the aggregation settlement of kaolinite particles. In particular, the understanding of aggregation characteristics of kaolinite particles in the presence of SO, Tween80 and alkylamine isn’t comprehensively discussed at present.

In this paper, effects and adsorption mechanism of three types of surfactants on the aggregation behavior of kaolinite particles were studied by the measurements of fractal dimension analysis, adsorption test, Infrared spectroscopy analysis and Zeta potential measurements. This study can provide a theoretical basis to develop new interface sorting methods for the fine kaolinite particles.

## Materials and methods

Kaolinite used in this work was collected from the Huaibei Jinyan Kaolinite Company (China). Firstly, it was purified in laboratory by eliminating the organic matters with H_2_O_2_, which was realized in a glass beaker with 30% H_2_O_2_/dry kaolinite samples for 24 h. Then, the slurry was kept in a water bath at 60°C to remove the remnant H_2_O_2_. The oxidized kaolinite particle was dispersed in 1 mol/dm^3^ NaCl solution with 2% solid concentration for 12 h and the Na^+^-kaolinite slurry was washed with pure water. The kaolinite samples were prepared by centrifugal treatment. Next, it was classified with 1000 mesh screen to obtain kaolinite particles of -13um and then put it aside for 12 h. The cleaned kaolinite particles were dried at room temperature as the sample in this study.

The kaolinite samples used in the experiment were analyzed using laser particle size analyzer (SALD-7101, Japan). Table 1 illustrated the size of the sample. It showed that d_50_ particle size of kaolinite sample was 11.78um. And the BET surface area of the sample was 13.224 m^2^/g.

**Table 1:** Particle size of kaolinite samples.

Sample	d_50_/um	d_75_/um
Kaolinite	11.78	15.78

Where d_50_ and d_75_ were the particle sizes corresponding to the cumulative particle size distribution of reaching 50% and 75%, respectively.

Where d_50_ and d_75_ were the particle sizes corresponding to the cumulative particle size distribution of reaching 50% and 75%, respectively.

**Fig 1:**
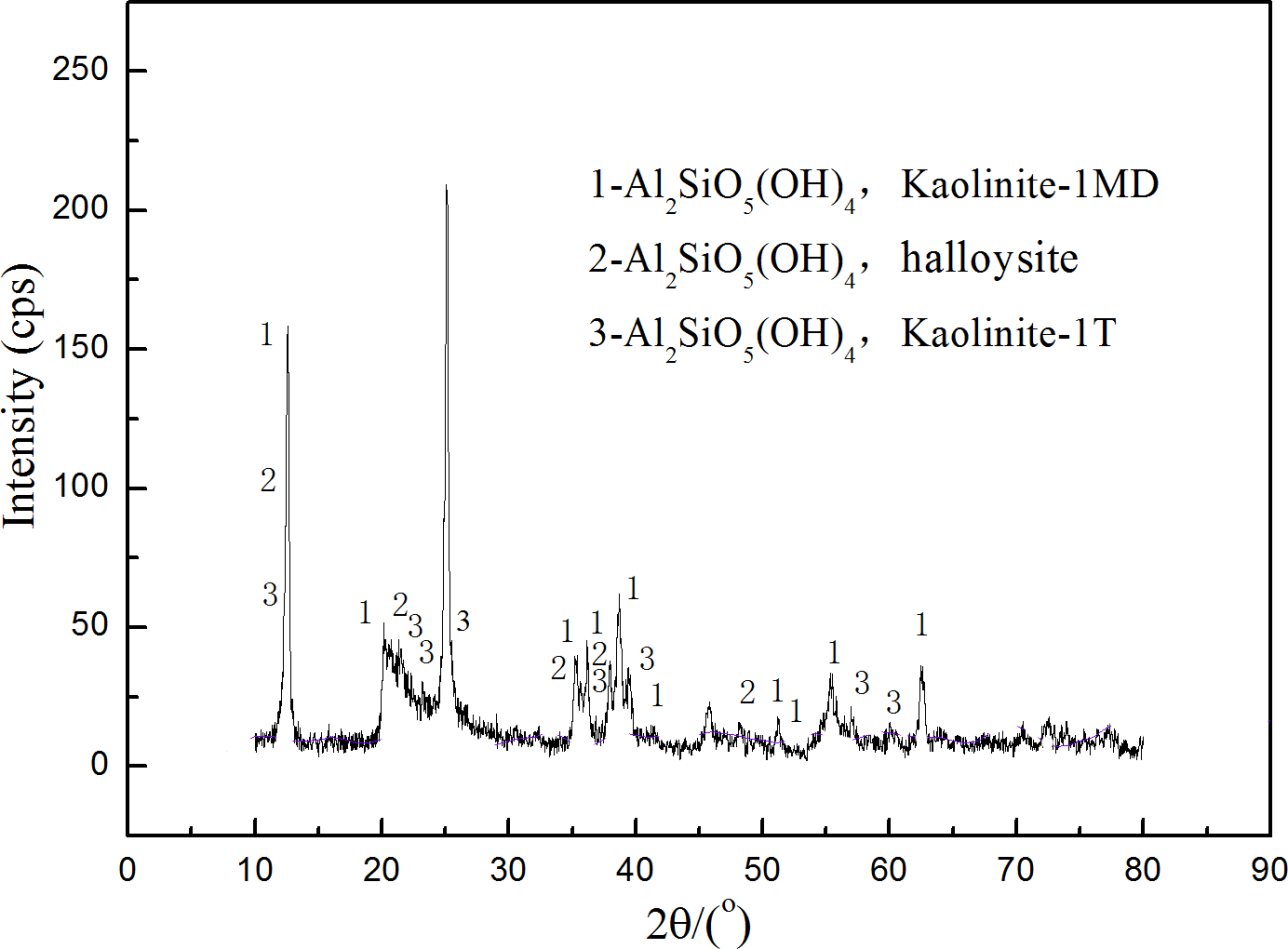
XRD pattern of kaolinite sample.

The mineral components were analyzed using X-ray diffraction meter (XRD-6000, Japan) with Cu Kα radiation. Fig 1 illustrated the X-ray diffraction pattern of the sample. It showed that there were three types of minerals, kaolinite-1MD, halloysite and kaolinite-1T. All of the three minerals belong to kaolinite family.

In this test, sodium oleate (SO), sodium dodecyl sulfate (SDS), sodium dodecyl benzene sulfonate (SDBS), dodecylamine (DDA), dodecyl trimethyl ammonium bromide (DTAB), cetyltrimethylammonium bromide (CTAB), Tween80, Span80 and Op10 used in this work were from Sinopharm Group Chemical Reagent Co. Ltd. (China). pH value of the aqueous solutions was adjusted by adding hydrochloric (HCl) or sodium hydroxide (NaOH). Sodium chloride (NaCl), sodium hydroxide (NaOH) and hydrochloric (HCl) were from Xilong Chemical Co. Ltd. (China). All of agents were analytical grade. The water was filtered through a resin layer with a pore size of 0.2 μm and the residual conductivity was less than 1 μS/cm.

### Settlement experiment

In this work, a measured dosage of kaolinite samples was first added into the water of 500 cm^3^ to prepare kaolinite suspension with 20 g/dm^3^ solid concentration. Then, the suspension was mixed immediately at the velocity of 500 rev/min with an electronic stirrer for 30 min. After that, the suspension was processed by ultrasonic disperser for 5 min with an ultrasonic homogenizer in order to completely wet the solid particles and ensure full dispersion of kaolinite particles.

A certain amount of surfactant was poured into the beaker. All pH values of the aqueous solutions were adjusted to the predetermined value by 1 mol/dm^3^ NaOH or HCl. The suspension was mixed immediately at a predetermined speed for a predetermined time. Then, the suspensions in the beaker were transferred to a 100 cm^3^ graduated cylinder, and precipitated in a 100 cm^3^ graduated cylinder for 7 min. The graduated cylinder was inverted for 3 times to make suspension homogeneous. Ultimately, the supernatant solution was withdrawn to be put into a quartz cell for the measurement of light transmittance and the remaining pulp filtration was dried, weighed and calculated the production rate of sedimentation. The settling yield was estimated by the following expression:

$$\omega =\frac{m}{M}\times 100\%$$ (1)

Where ω was the settling yield,%; m was the weight of sedimentation after settlement, g; M was the total sample weight used for making sedimentation experiment, g.

### Determination of adsorption capacity

The adsorption capacity of cationic surfactants on mineral surface was determined by a new two-phase titration method [17, 18].

The residual concentration method was used to determine the adsorption capacity, which was calculated by the following formula [12]:

$$\Gamma =\frac{(C0-C)V}{mA}\times 100\%$$ (2)

Where Γ was adsorption (mol/m^2^); C_o_, C were the initial and final concentrations, respectively, (mol/dm^3^); V was the volume of solution (dm^3^); m was the quality of sample (g); A was the specific surface area of ore sample (m^2^/g).

The adsorption of surfactants on mineral surface was determined by the spectrophotometric method [19]. The standard curves and the measured dates were presented in S1 File.

### Zeta potential measurement

A certain amount of kaolinite samples was prepared for the mixture suspension of the mass fraction of 1%. The zeta potentials were measured by a Zeta probe zeta meter (USA), at 25°C, using the automatic titration method and the average of three measurements was taken in the present results.

### Infrared spectroscopy analysis

Samples for infrared spectroscopy analysis were prepared by mixing 300 mg KBr with 3 mg kaolinite. The d_50_ of kaolinite particles was less than 2 μm by grinding in an agate mortar. Then, the samples were pressed at a pressure of 30-40 MPa. The infrared spectroscopy analysis was obtained by a NICOLET-380 Fourier Infrared Spectrometer (USA). The scanning range of apparatus was 400~4000 cm^-1^.

### Fractal characteristics of the flocculation

The kaolinite suspension with surfactants was used for the analysis of flocculation fractal characteristics. pH value was adjusted by 1 mol/dm^3^ NaOH or HCl solution. The suspension were firstly added into a beaker and stirred at 750 rev/min for 30 min. and then the suspension was placed into a 100 cm^3^ cylinder for 7 min. The flocs were taken out at the same position by a dropper and placed on a glass slides. The appearance of sediments and microstructure was observed using a scanning electron microscope (S3000-N, Japan).

## Results and discussion

### Effect of surfactant on settlement characteristics of kaolinite particle

**Fig 2:**
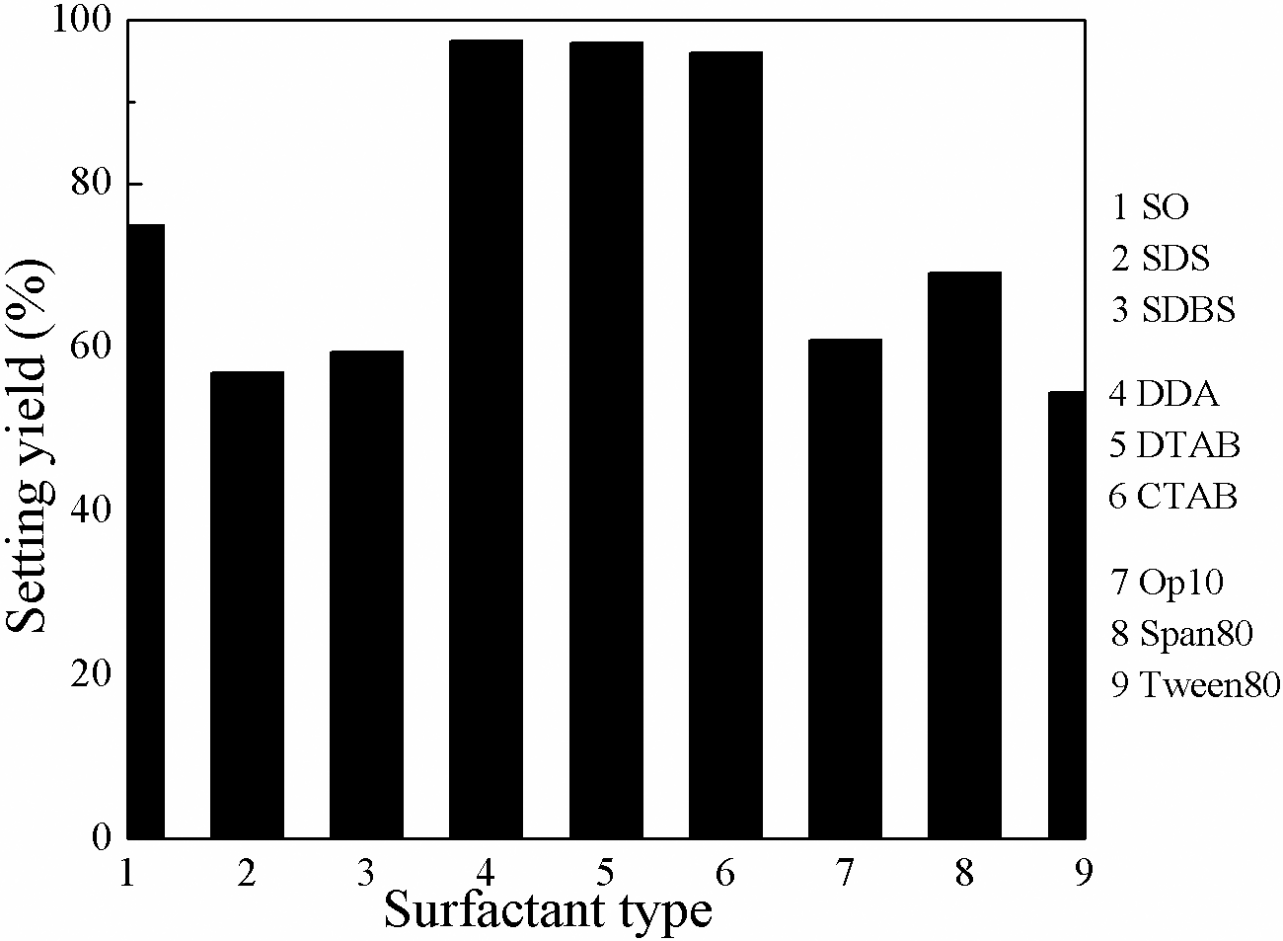
Settling yield of kaolinite as a function of surfactant type.

As shown in Fig 2 that 97.5% settling yield of kaolinite was obtained in the presence of cationic surfactants. While only 74.9% settling yield was obtained in the presence of anionic surfactants. And for the nonionic surfactants, the settling yields were lower than that of both cationic and anionic surfactants.

**Fig 3:**
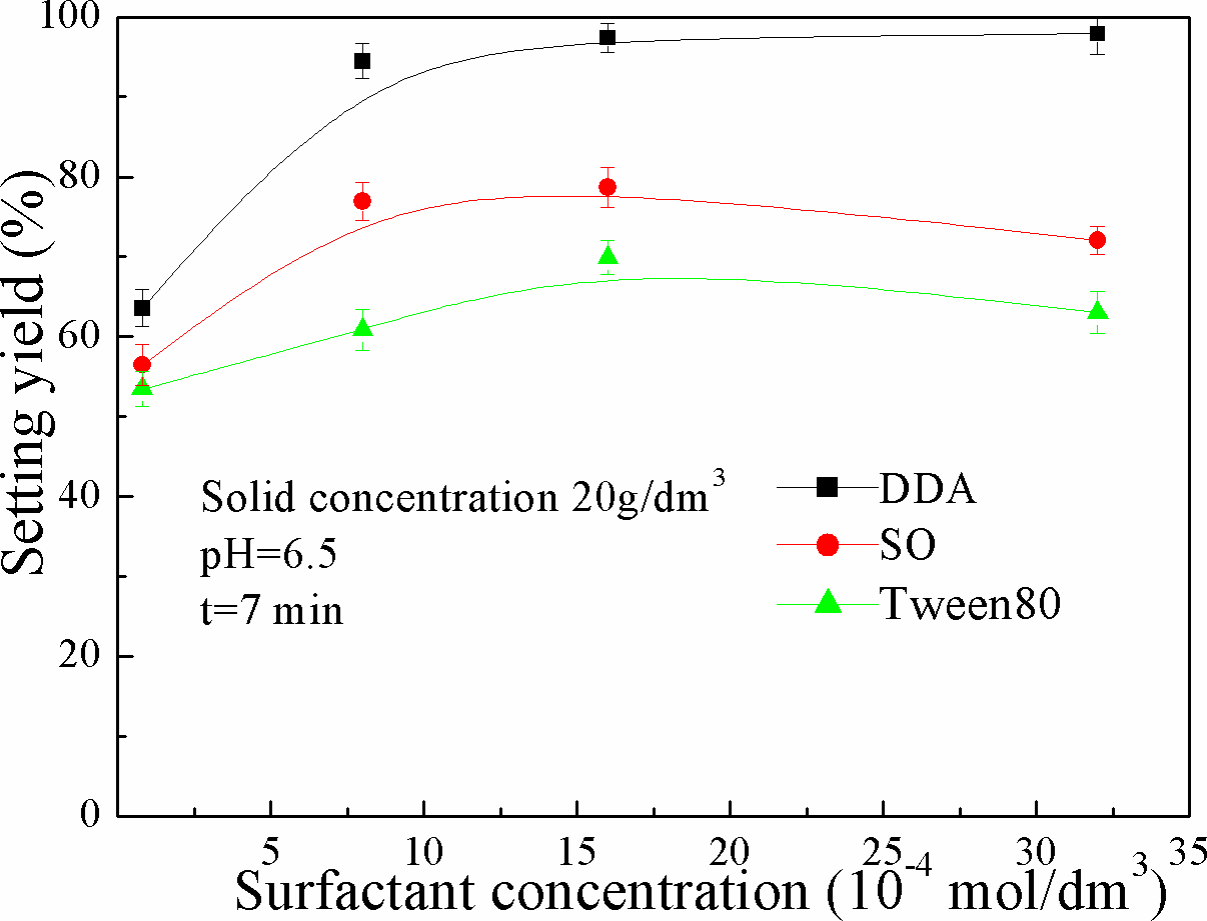
Settling yield of kaolinite as a function of surfactant concentration (The data points represent the mean values (n = 3) ± standard deviation).

The settling yield of kaolinite as a function of surfactant concentration was presented in Fig 3. As shown in Fig 3, the settling yield of kaolinite particles increased rapidly when the DDA concentration increased from 0.8×10^-4^ to 8×10^-4^ and 98% settling yield was obtained in the presence of 3.2×10-3 mol/dm3 DDA concentration. The curve of relationship gently changed, which indicated that the change of DDA concentration had little effect on the settling yield of kaolinite. When the concentration of SO gradually increased from 0 to 1.0×10^-3^ mol/dm^3^, the settling yield significantly increased from 55% to 78%. The settling yield reached the maximum value when the concentration of SO reached a certain value (1.0×10^-3^ mol/dm^3^). When Tween 80 was added into the kaolinite suspension, the settling yield of kaolinite was similar to that in the presence of SO. However, when the concentration of SO and Tween80 were more than 1.0×10^-3^ mol/dm^3^ and 1.6×10^-3^ mol/dm^3^, respectively, the settling yield of kaolinite decreased with the increase of the surfactant concentration.

### Effect of surfactant type and concentration on fractal dimension of kaolinite floc

The surface and interior of flocs formed by aggregating have self-similarity and scale invariance, which have fractal characteristics. The fractal dimension of the floc can describe the flocculation structure and its complexity to a certain extent, which can reflect the positive effect of flocculation and sedimentation. Meanwhile, the fractal dimension can be used to reflect the effect of flocculation and regarded as an index.

Table 2 illustrated the fractal dimension of kaolinite particles as a function of surfactant type and concentration at pH=6.5. When the concentration of DDA varied from 8.0×10^-4^ mol/dm^3^ to 3.2×10^-3^ mol/dm^3^, the fractal dimension of kaolinite was more than 1.70, however, floc fractal dimension values changed little. As the SO concentration increased further, the fractal dimension decreased to the minimum value at 1.0×10^-3^ mol/dm^3^, and then increased with the increase of SO concentration. With the increase of Tween80 concentration, the trend of fractal dimension was similar to that of SO, but the fractal dimension reached the minimum value at 1.6×10^-3^ mol/dm^3^.

**Table 2:** Fractal dimension of flocculating as a function of surfactant type and concentration (pH=6.5).

Surfactant	Surfactant concentration (mol/dm^3^)		
	8.0×10^-4^	1.6×10^-3^	3.2×10^-3^
DDA	1.71	1.70	1.72
SO	1.33	1.68	1.49
Tween80	1.11	1.56	1.03

In contrast to Fig 3 and Table 2, it can be seen that the change of surfactant type and concentration had obvious agreement on the influence of settling yield and floc fractal dimension of kaolinite particles. The effect of DDA on the settling yield and floc fractal dimension of kaolinite particles was small, and the floc fractal dimension of adding Tween80 was higher than that of SO.

**Fig 4:**
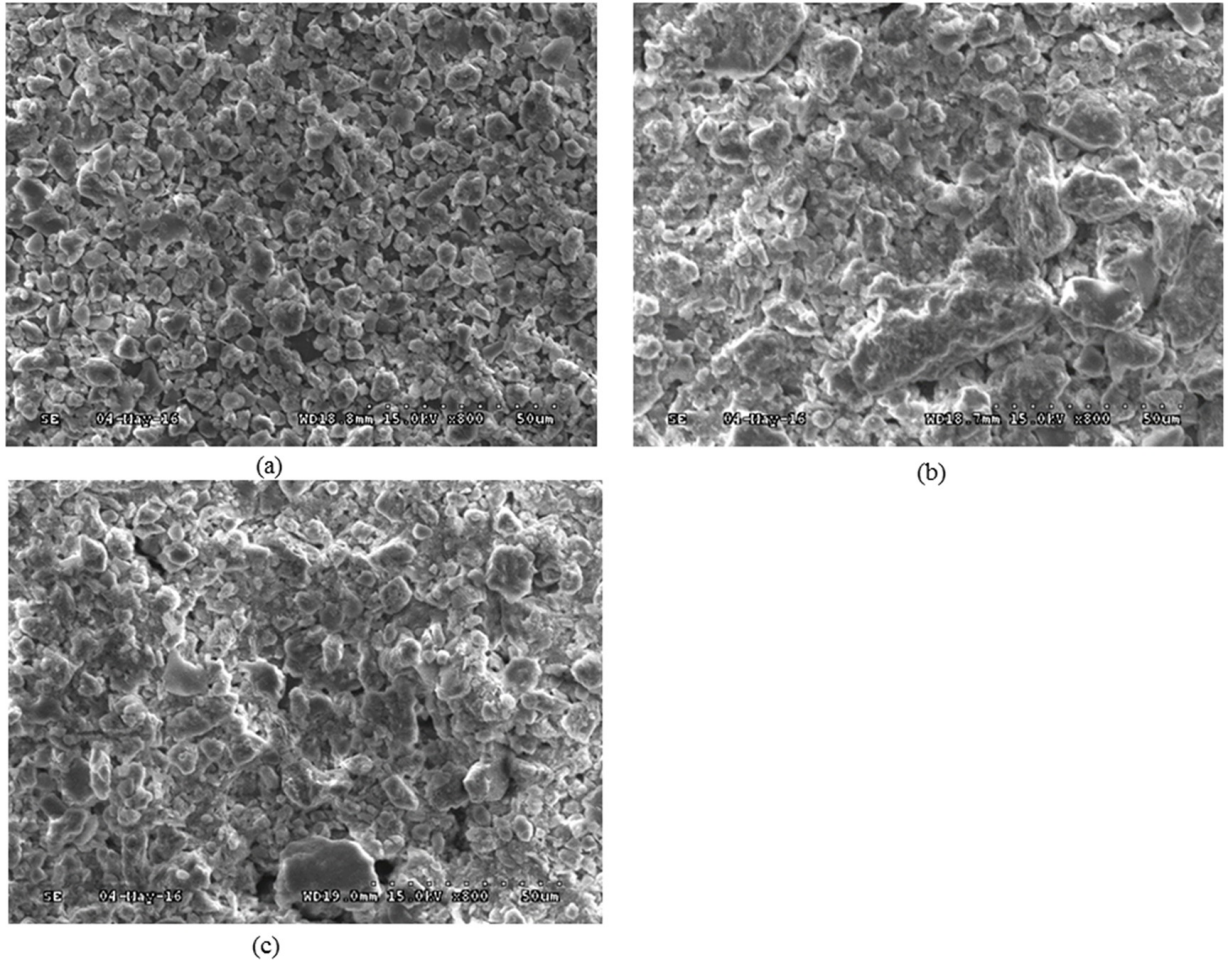
SEM photographs of aggregation performance under different Tween80 concentration (a) 8.0×10^-4^ mol/dm^3^ (b) 1.6×10^-3^ mol/dm^3^ (c) 3.2×10^-3^ mol/dm^3^.

**Fig 5:**
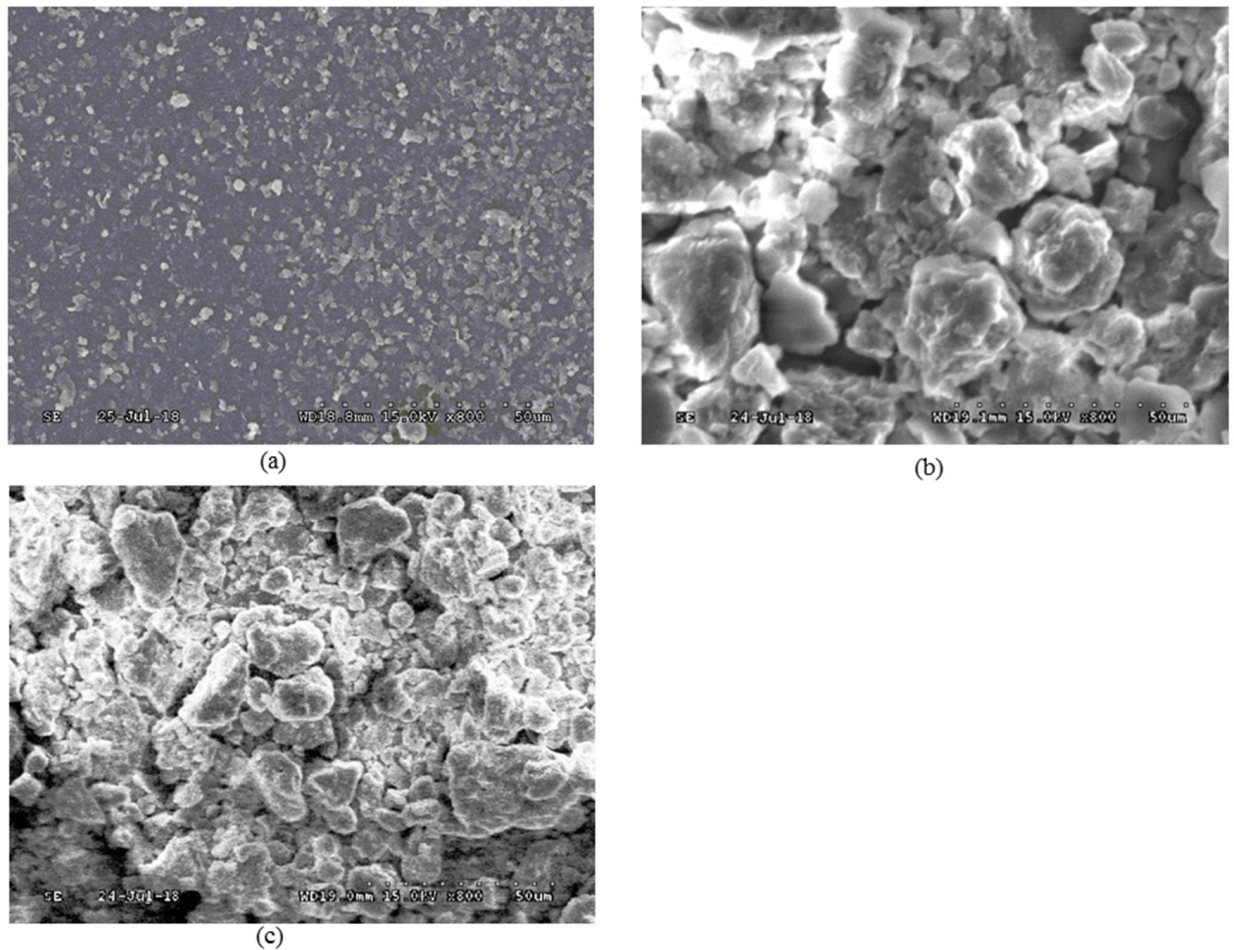
SEM photograph of kaolinite aggregation performance under different surfactant (a) Kaolinite (b) Kaolinite+DDA (c) Kaolinite+SO (pH 6.5, Surfactant concentration 1.6×10^-3^ mol/dm^3^).

SEM photographs of aggregation performance of kaolinite particles in the presence of different surfactant concentration and surfactant type were presented in Fig 4 and Fig 5. It can be seen from Fig 4 that the floccules size and volume gradually went up with the increase of Tween80 concentration and then gradually went down to be low with Tween80 concentration higher than 1.6×10^-3^ mol/dm^3^.

As shown in Fig 5(A) that kaolinite particles without added surfactant showed a highly dispersed state in water and the particle size were less than 5um. When DDA was added, many flocs occurred as presented in Fig 5(B), which indicated that DDA can significantly promote the aggregation of kaolinite particles.

### Effect of pH on sedimentation of kaolinite particles

**Fig 6:**
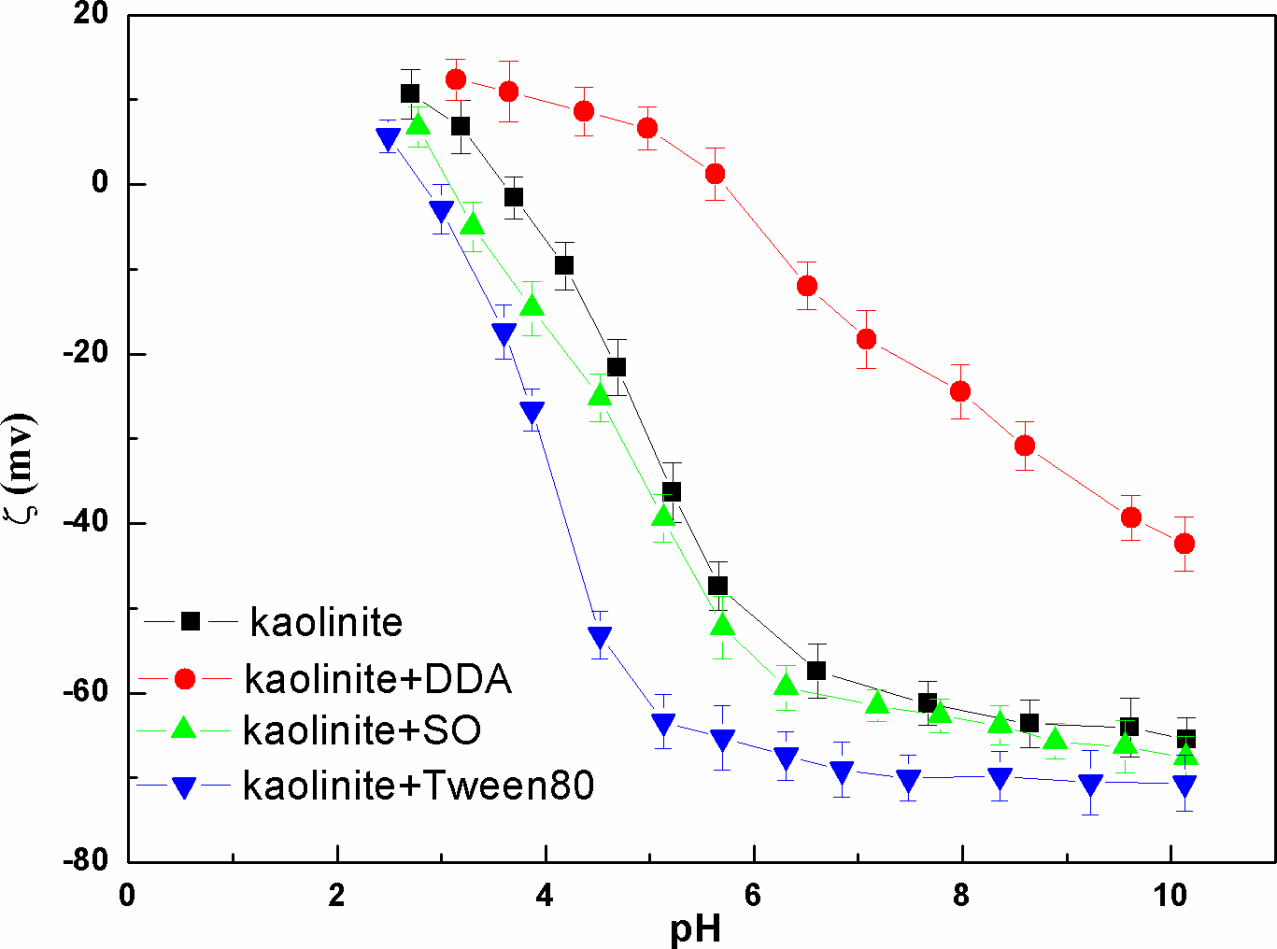
Zeta potential of kaolinite surface as a function of pH (The data points represent the mean values (n = 3) ± standard deviation).

Zeta potential of kaolinite surface as a function of pH value was presented in Fig 6. It can be seen that the zeta potential of kaolinite in the presence of DDA was closer to the 0 mv. The electrostatic repulsion between kaolinite particles was decreased, which was beneficial for the kaolinite particles aggregation.

**Fig 7:**
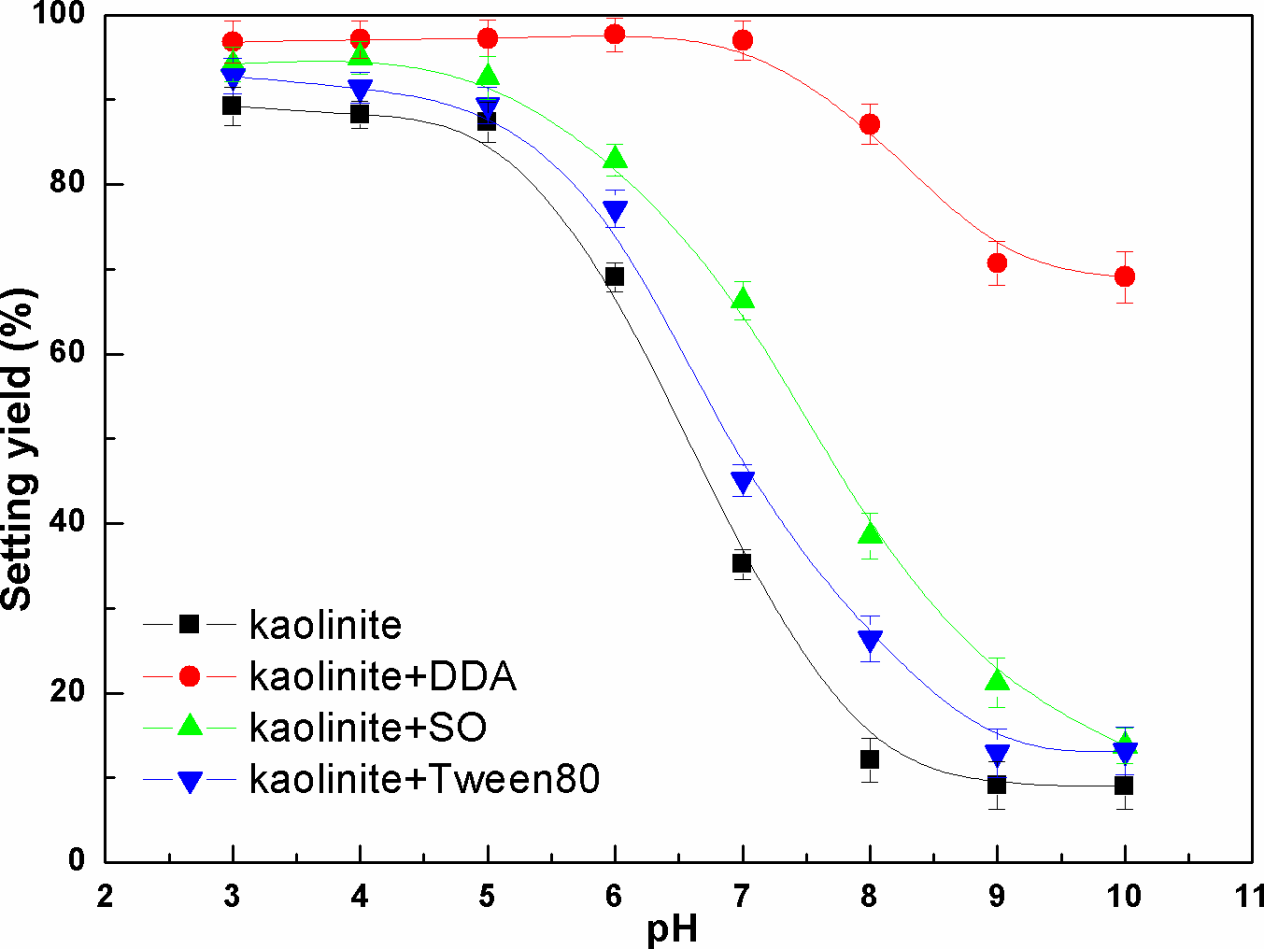
Setting yield of kaolinite as a function of pH (The data points represent the mean values (n = 3) ± standard deviation).

Effect of pH on the setting yield of kaolinite particles was showed in Fig 7. As shown in Fig 7 the setting yields of kaolinite particles decreased with the increase of the pH value. However, the setting yields of kaolinite+DDA was the highest in all pH ranges. 98% setting yield was obtained with kaolinite+DDA at pH=7 compared with that of only 66%, 45% and 35% in the presence of other surfactants.

### Sedimentation mechanism of kaolinite particles in different surfactant solutions

**Fig 8:**
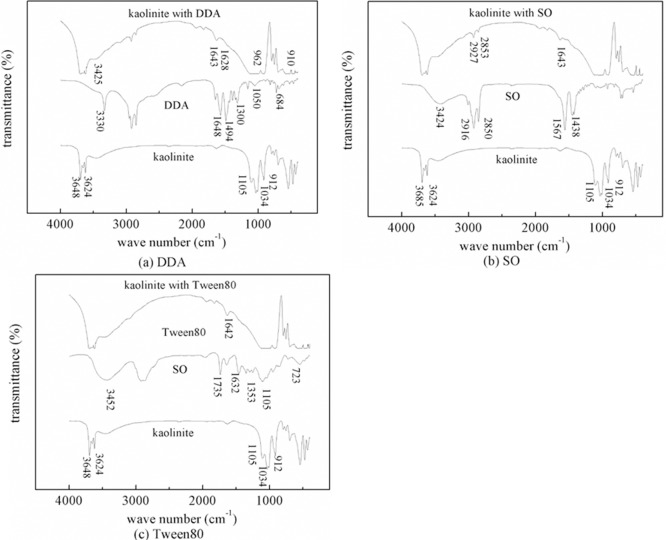
FTIR spectra of kaolinite untreated and treated by 3.2×10^-3^ mol/dm^3^ (a) DDA (b) SO and (c) Tween80.

FTIR spectra of kaolinite conditioned with 3.2×10^-3^ mol/dm^3^ surfactant solutions were presented in Fig 8. As shown in Fig 8(A) FTIR spectra of kaolinite treated with DDA showed absorptions at 1628 cm^-1^. This situation is attributed to stretching vibrations of N-H in the amine salt, which suggested that DDA could be adsorbed to the surface of kaolinite particles. As a result of the high electronegativity of hydrogen bonds and oxygen atoms, the N-H stretching absorption peak of the dodecamine molecule at 3330 cm^-1^ drifted up to 3425 cm^-1^. It revealed that DDA was adsorbed to the kaolinite particles by electrostatic interaction and hydrogen bonding.

**Fig 9:**
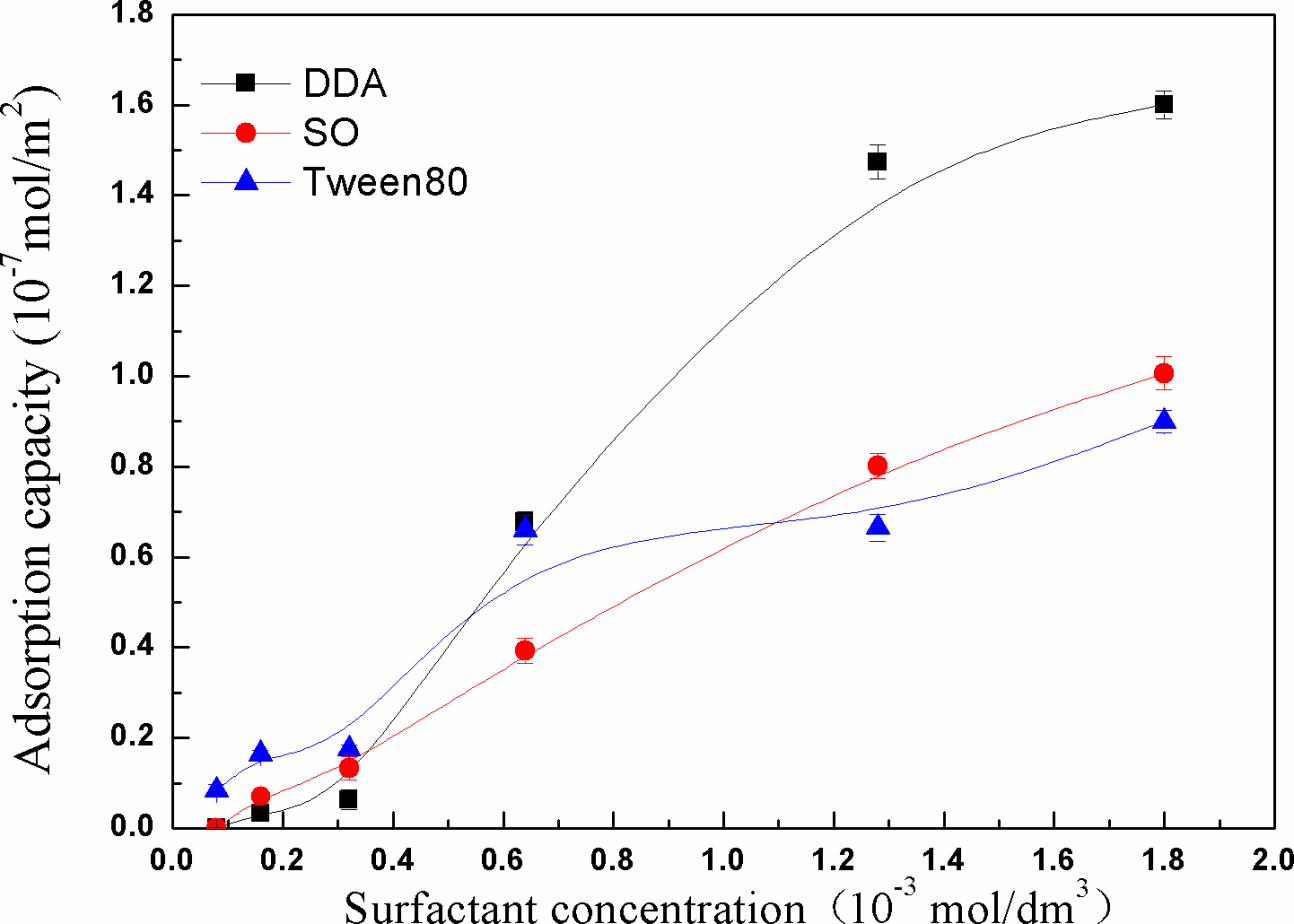
Adsorption capacity of DDA, SO and Tween80 as a function of surfactant concentration.

Many scholars have found that the adsorption of surfactants on kaolinite surface is an important factor, which has great influence on the stability of colloidal dispersions [11]. Due to the different adsorption manner, the adsorption capacity of surfactants on kaolinite surface was also different. Therefore, it is indispensable to study the changes of the adsorption capacity of different surfactants on kaolinite surface. Fig 9 showed the change of the adsorption of DDA on kaolinite surface. It was found that the adsorption capacity of DDA on kaolinite surface increased with the increase of DDA concentration. When the concentration of DDA was 1.6×10^-3^ mol/dm^3^, the adsorption capacity increased slightly, but the adsorption capacity was still larger than that of SO and Tween80. After the -NH_2_ group is adsorbed, the particles are liable to react with the other charged negative particles. Meanwhile, due to the neutralization of the electric charge, the zeta potentials of the negative charged particles are significantly reduced. The kaolinite particles strongly aggregated with DDA by Van der Waals attraction in the energy input as a result of the decreased electrostatic repulsion, which was in line with the classic Derjaguin-Landau-Verwey-Overbeek (DLVO) theory. Due to the DDA preferential adsorption on kaolinite tetrahedral surface, the hydrophilicity and hydrophobicity of kaolinite surface are different, resulting in the hydrophobic flocculation of the particles under the influence of the hydraulic force [20]. At the same time, the solitary electrons of the nitrogen atoms in the amine molecules can be shared with the surface metal cations of the kaolinite minerals. Accordingly, the relatively stable complexes are formed by covalent bond. That is, the amine molecules can produce complex adsorption on the mineral surface to make the mineral surface hydrophobic, which result in the occurrence of hydrophobic aggregation settlement. Thereby, with the addition of DDA, the settling yield and floc fractal dimension increased.

In Fig 8(B), FTIR spectra of kaolinite treated with SO showed absorptions at 2853 cm^-1^ and 2927 cm^-1^. Comparing with the stretching vibration absorption peaks at 2850 cm^-1^ and 2916 cm^-1^, the wave number of CH_3_, (CH_2_) shifted by 3 cm^-1^ and 11 cm^-1^, respectively, comparing with the SO infrared spectrum. Because of the stretching vibration of the silicic bonds exposed to the kaolinite surface, the flexural vibration of siloxane bonds is shifted and the vibration is weak in the 380~550 cm^-1^ region. The absorption peak which was generated by the aluminum oxide vibration changed at 912 cm^-1^. The reason may be that the Al surface of the mineral surface which was chemically adsorbed with the carboxylic acid groups of oleic acid can change the absorption peak. All of the above showed that SO was chemically adsorbed on kaolinite surface [21]. However, it can be formed by double adsorption of alkane hydrophobic bonds.

Fig 9 showed that the adsorption amount of surfactants on kaolinite surface varied markedly with the increase of concentration. When the concentration was higher than 8.0×10^-4^ mol/dm^3^, the adsorption structure of the interface between kaolinite and SO was mainly monomolecular layer. The nonpolar base facing the solution can increase and accumulate a number of solute molecules on kaolinite surface. The hydrophobicity of the kaolinite particles increased, and the kaolinite particles strongly aggregated as a result of the bridging function of nonpolar groups, resulting in a high sedimentation yield. When the concentration was greater than the critical micelle concentration (1.0×10^-3^ mol/dm^3^), the electrostatic repulsion between positively charged micelles prevents the non-polar from bridging effect, which enhances the dispersion of kaolinite particles and decreases the settling yield of kaolinite particles.

Some scholars believed that the solubility of SO is 10^-7.6^ mol/dm^3^ in an aqueous solution. In kaolinite suspension, the oleic acid concentration in the pulp is larger than its solubility. According to the equilibrium reaction equation, the relationship is obtained between the concentration of each component in aqueous acid solution and pH value. When the suspension is accompanied by SO, RCOOH_(l)_ RCOOH_(aq)_, RCOOH•RCOO-, RCOO- and RCOO_2_^2-^, the concentration of each component is closely related to pH. At a certain concentration, oleic acid is mainly present in an ionic state, and there are also RCOOH•RCOO- in the presence of an ion-molecule association at pH = 7. The latter is easier to react chemically with Al^3+^ on kaolinite surface to make the hydrophobic particles form aggregates [22, 23].

**Fig 10:**
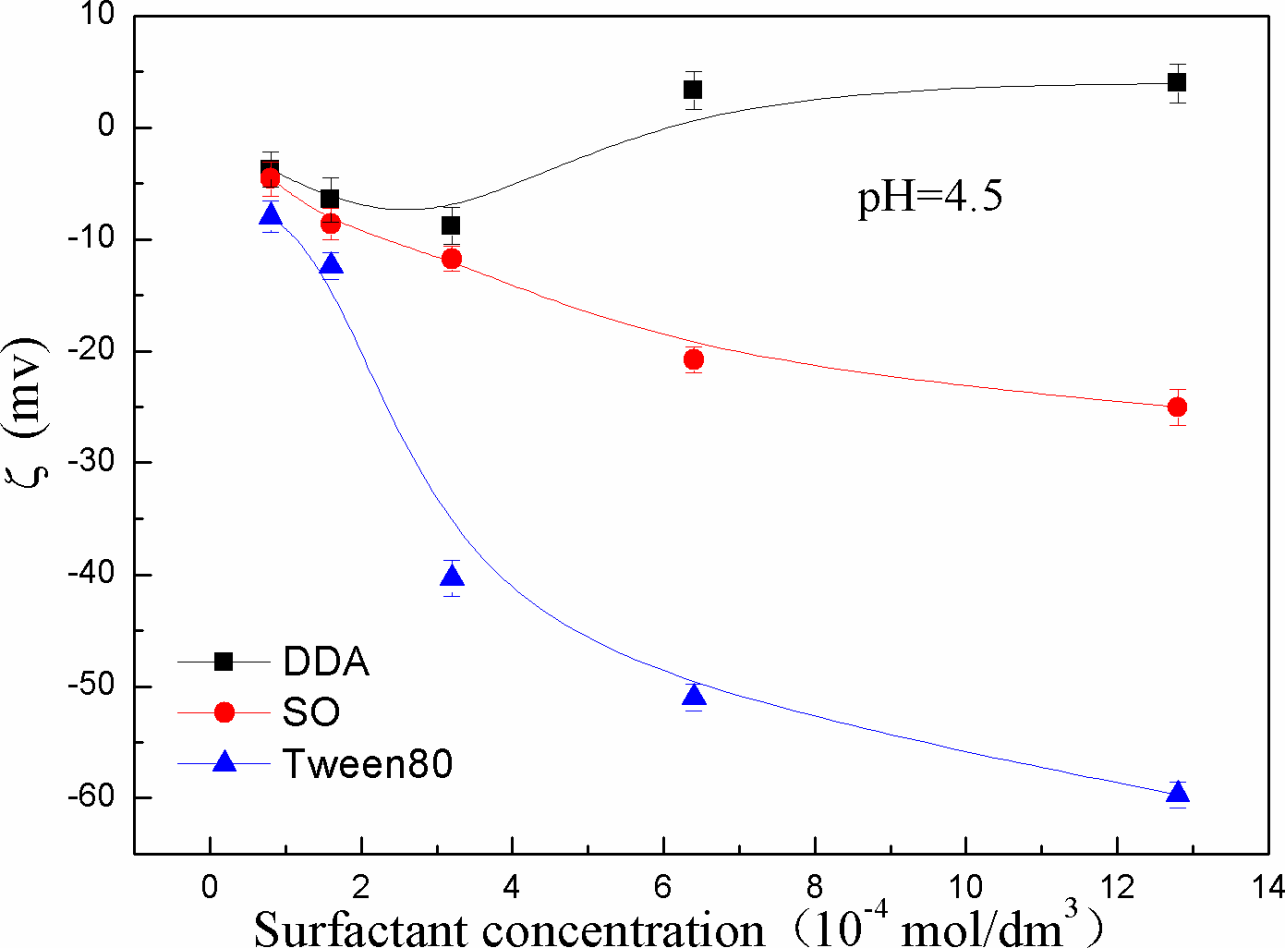
Effect of DDA, SO and Tween80 on zeta potential of kaolinite surface.

Because of the low content of RCOOH•RCOO- component, the settling yield and floc fractal dimension of kaolinite induced by SO are weaker than that of DDA. In addition, Fig 10 illustrated the electric potential of the kaolinite particles surface decreased when a certain amount of SO was added into the kaolinite suspension. Since kaolinite is a layered silicate mineral, it is easy to be cleaved along the surface of the silica or tetrahedral surface. Another surface is more difficult to be broken. Therefore, the Al^3+^ active point on kaolinite surface is relatively little, which is not conducive to the adsorption of oleate. The reason that the settling yield and the fractal dimension of adding SO were smaller than that of DDA and greater than of Tween80 was that the change of the surface electric potential was limited.

FTIR spectra of kaolinite treated with Tween80 showed absorptions in Fig 8(C). The absorption peaks of C-O-C and C=O of Tween80 do not appear in the FTIR spectra of kaolinite, indicating that Tween80 was hardly adsorbed to the kaolinite surface. The reason was that Tween80 must be replaced by enough water molecules on solid interface when Tween80 is adsorbed to the oxide surface. Unfortunately, Tween80 can’t replace the above water molecules after hydration, so it is difficult to adsorb on kaolinite surface [24]. In addition, Fig 8(C) illustrated that the less than 1000 cm^-1^ peak changed after surfactants were adsorbed on the kaolinite surface. These locations are usually attributed to OH characteristic frequency, which makes the spectrum complex in the 700~950 cm^-1^ region. At the same time, new bands in the 800~1200 cm^-1^ region for kaolinite with Tween80 are attributed to the C-C stretching vibration, which is weak and susceptible to vibration coupling.

**Fig 11:**
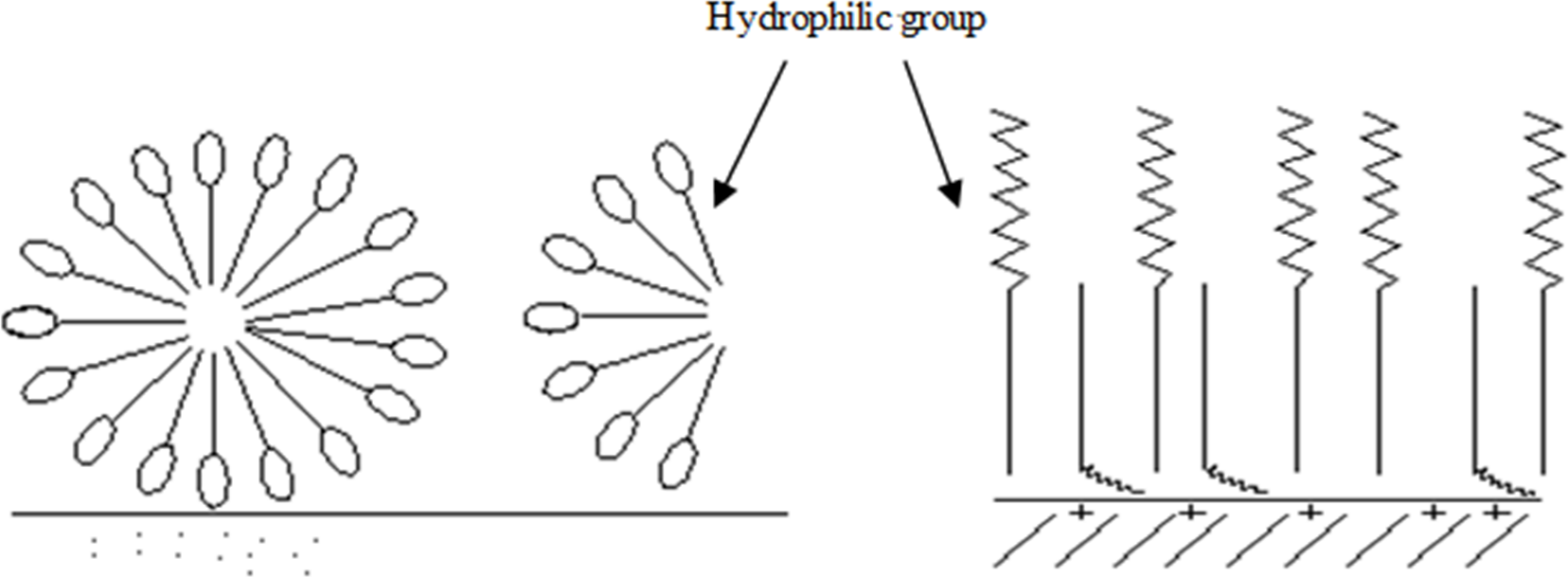
Adsorption of nonionic surfactants on kaolinite surface.

Tween80 was hardly to be adsorbed on the kaolinite surface as a result of being not ionized in aqueous solution. Although the adsorption capacity was low, it changed with the increase of Tween80 concentration. As the concentration continued to increase, the interaction between the hydrophilic group and the particle surface was weak. In spite of the strong hydrophobic and the hydrophilic base facing water molecules, the hydrophobic base lied on the interface. Meanwhile, the interface was covered by Tween 80 molecules, and the adsorption capacity reached equilibrium. Fig 11 showed that Tween80 began to form a large number of micelles in the solution when the concentration of Tween80 was up to 6.0×10^-4^ mol/dm^3^, and the change of adsorption capacity continued to increase. However, as described in Fig 11, some nonionic surfactant molecules were adsorbed to the interface in the opposite direction, and the hydrophilic chains were suspended in aqueous solution, which rendered the hydrophobic kaolinite surface re-hydrophilic and created a space for the adjacent particles resistance. Although Tween80 is difficult to be adsorbed on the kaolinite surface, the hydrophilic group of Tween80 is mainly composed of a certain number of oxygen-containing functional groups. Therefore, with the increase of Tween80 concentration, the Zeta potential of kaolinite surface decreased. Moreover, the change of Zeta potential was obvious, which resulted in the minimum sediment yield and floc fractal dimension. This was consistent with the experimental results (see Fig 3).

It can be seen that a certain amount of surfactant can reduce the potential energy of the electrostatic repulsion between the particles, so the energy barrier of being overcome between the particles was also reduced, resulting in making the particles form aggregates. However, due to the different types and concentrations of surfactants, the surface electrical potentials of kaolinite particles were different, which resulted in the corresponding changes of the potential of electrostatic repulsion forces. Therefore, it has different effects on the interglacial settling characteristics.

## Conclusions

1. Cationic surfactants showed the best performance in promoting the kaolinite aggregation compared with anionic and non-ionic surfactants. The settling yield of kaolinite particles was more than 98%, and the fractal dimension of the flocs was up to 1.70 in the presence of DDA.
2. The adsorption capacity of DDA on kaolinite surface was larger than that of SO and Tween80. DDA can adsorb on kaolinite surface both by electrostatic and hydrogen-bonding interactions. While SO was adsorbed by chemical adsorption and Tween80 was hardly to be adsorbed. The electronegativity of the fine kaolinite particles increased with the increasing of the SO and Tween80 concentration, which was detrimental to the aggregation settlement of kaolinite.

## Supporting information

S1 File Original dates of the adsorption experiments. (DOCX)

S1 Table XRD pattern of kaolinite sample. (XLSX)

S2 Table Settling yield of kaolinite as a function of surfactant type. (XLSX)

S3 Table Settling yield of kaolinite as a function of surfactant concentration. (XLSX)

S4 Table Zeta potential of kaolinite surface as a function of pH. (XLSX)

S5 Table Setting yield of kaolinite as a function of pH. (XLSX)

S6 Table FTIR spectra of kaolinite untreated and treated by 3.2×10-3 mol/dm3 DDA, SO and Tween80. (XLSX)

S7 Table Adsorption capacity of DDA, SO and Tween80 as a function of surfactant concentration. (XLSX)

S8 Table Effect of DDA, SO and Tween80 on zeta potential of kaolinite surface. (XLSX)
