# Supplementary material for: Study on the aggregation behavior of kaolinite particles in the presence of cationic, anionic and non-ionic surfactants
Source: PLoS One. 2018 Sep 13;13(9):e0204037. doi: 10.1371/journal.pone.0204037 (PMC6136786; doi:10.1371/journal.pone.0204037)
Supplement: S1 File — (DOCX) [file pone.0204037.s001.docx]

The adsorption amounts of DDA, SO and Tween80 on kaolinite were determined by spectrophotometry. Eosine Y, Nile blue and KI_2_-I_2_ were used as chromogenic agent for DDA, SO and Tween80, respectively. The standard curves of the surfactants were presented in Fig1, Fig2, and Fig3. And the original dates were presented in Table1, Table3 and Table5. The dates of the final concentration of the surfactants in the adsorption experiments were presented in Table2, Table4 and Table6.





Fig.1 Standard curve of DDA

Table 1 Original dates of DDA standard curve

| Initial concentration (mg/dm^3^) | A (abs) |
| --- | --- |
|  |  |
| 0 | 0 |
| 2 | 0.097 |
| 4 | 0.238 |
| 6 | 0.351 |
| 8 | 0.462 |
| 10 | 0.586 |

Table 2 Original dates of DDA adsorption experiment

|  | Final concentrations (10^-4^mol/dm^3^) | | |
| --- | --- | --- | --- |
| Initial concentration  （10^-3^ mol/dm^3^） | 1# | 2# | 3# |
|  |  |  |  |
| 0.08 | 0.79979 | 0.79995 | 0.79974 |
| 0.16 | 1.59126 | 1.59674 | 1.58598 |
| 0.32 | 3.18249 | 3.19089 | 3.17692 |
| 0.64 | 6.22277 | 6.22823 | 6.21348 |
| 1.28 | 12.41272 | 12.39983 | 12.42399 |
| 1.8 | 17.57990 | 17.58820 | 17.56851 |





Fig.2 Standard curve of SO

Table 3 Original dates of SO standard curve

| Initial concentration (mg/dm^3^) | A (abs) |
| --- | --- |
|  |  |
| 0 | 0 |
| 2 | 0.0212 |
| 4 | 0.0376 |
| 6 | 0.0567 |
| 8 | 0.0783 |
| 10 | 0.0964 |

Table 4 Original dates of SO adsorption experiment

|  | Final concentrations (10^-4^mol/dm^3^) | | |
| --- | --- | --- | --- |
| Initial concentration  （10^-3^ mol/dm^3^） | 1# | 2# | 3# |
|  |  |  |  |
| 0.08 | 0.80000 | 0.79984 | 0.79989 |
| 0.16 | 1.58146 | 1.58428 | 1.57868 |
| 0.32 | 3.16479 | 3.17319 | 3.15710 |
| 0.64 | 6.29600 | 6.30642 | 6.28839 |
| 1.28 | 12.58906 | 12.59675 | 12.57882 |
| 1.8 | 17.73744 | 17.72604 | 17.74949 |





Fig.3 Standard curve of Tween80

Table 5 Original dates of Tween80 standard curve

| Initial concentration (mg/dm^3^) | A (abs) |
| --- | --- |
|  |  |
| 0 | 0 |
| 2 | 0.035 |
| 4 | 0.067 |
| 6 | 0.079 |
| 8 | 0.124 |
| 10 | 0.153 |

Table 6 Original dates of SO adsorption experiment

|  | Final concentrations (10^-4^mol/dm^3^) | | |
| --- | --- | --- | --- |
| Initial concentration  （10^-3^ mol/dm^3^） | 1# | 2# | 3# |
|  |  |  |  |
| 0.08 | 0.77378 | 0.78195 | 0.77847 |
| 0.16 | 1.55481 | 1.55999 | 1.55594 |
| 0.32 | 3.15649 | 3.15080 | 3.15471 |
| 0.64 | 6.23685 | 6.21558 | 6.22755 |
| 1.28 | 12.61655 | 12.63614 | 12.62650 |
| 1.8 | 17.75755 | 17.77310 | 17.76370 |
